# Rapid and Recent World-Wide Diversification of Bluegrasses (*Poa*, Poaceae) and Related Genera

**DOI:** 10.1371/journal.pone.0060061

**Published:** 2013-03-27

**Authors:** Matthias H. Hoffmann, Julia Schneider, Philipp Hase, Martin Röser

**Affiliations:** Martin Luther University Halle-Wittenberg, Institute of Biology, Geobotany and Botanical Garden, Halle, Germany; University of Lausanne, Switzerland

## Abstract

Rapid species diversifications provide fascinating insight into the development of biodiversity in time and space. Most biological radiations studied to date, for example that of cichlid fishes or Andean lupines, are confined to isolated geographical areas like lakes, islands or island-like regions. Using DNA sequence data of the ribosomal internal transcribed spacer (ITS) for many species of the *Poa* alliance, a group comprising about 775 C_3_ grass species, revealed rapid and parallel diversifications in various parts of the world. Some of these radiations are restricted to isolated areas like the Andes, whereas others are typical of the lowlands of mainly the northern hemisphere. These radiations thus are not restricted to island-like areas and are seemingly actively ongoing. The ages of the diversifying clades are estimated to be 2.5–0.23 million years (Myr). Conservative diversification rates in the *Poa* alliance amount to 0.89–3.14 species per Myr, thus are in the order of, or even exceeding, other instances of well-known radiations. The grass radiations of the mainly cold-adapted *Poa* alliance coincide with the Late Tertiary global cooling, which resulted in the retreat of forests and the subsequent formation of cold-adapted grasslands especially in the northern, but also in parts of the southern hemisphere. The cold tolerance, suggested to be one of the ecological key innovations, may have been acquired during the early diversification of the subfamily Pooideae, but became significant millions of years later during the Pliocene/Pleistocene radiation of the *Poa* alliance.

## Introduction

Plant diversification, i.e. the evolutionary origin of new species, genera and families, proceeds at different speeds. Net diversification of families and genera was estimated as 0.078 to 0.09 per Myr [1]. Species-level diversification exceeds these rates largely and were reported to be up to 7.6 new species per million years in Eurasian *Dianthus* species [2]. Other examples of rapid radiations were found, for example, in the genus *Lupinus*, in the Central and South American genus *Costus* or in South African ice plants [3]–[6], summarized in [2]. The magnitudes of these diversification rates are comparable to the famous radiation of cichlid fishes in east African rift lakes [7]. A common feature of these radiations is that they often occur on islands or island-like habitats, like lakes or mountain systems [2], [5], [8], [9]. Such areas of radiations frequently coincide with areas of overall high biodiversity [10]. Most parts of the earth, however, are not island-like, but consist of continental lowlands, in which biological radiations are seemingly rare. Examples of radiations in such regions have been reported only occasionally, for instance for plants in South Africa with the peculiar arid winter rainfall climate [3], [11] or the Mediterranean basin and adjacent mountains of the Irano-Turanian region [2]. The genus *Carex* provides another example of rapid diversification in non-island like areas [12] with diversification rates of up to 1.75 species per Myr. This number might be higher, if more sequences, particularly of so-called ‘difficult groups’ would be analysed. Here we report on a striking example of rapid diversification, which is not confined to islands or island-like areas, but proceeds with high speciation rate on all continents with the exception of the Antarctic. Bluegrasses (*Poa* L.) with about 500 species have almost world-wide distribution. Chloroplast and nuclear DNA studies revealed a well-supported clade of *Poa* and allied genera in the phylogeny of grasses (PPAM clade, i.e. Puccinelliinae/Coleanthinae, Poinae, Alopecurinae, and Miliinae in the sense of [13]), in the following termed *Poa* alliance [13]–[16]. The species of the *Poa* alliance are C_3_ grasses, which have their main distribution in the colder zones of the Earth and are not present or abundant in tropical and subtropical savannas [17]. Species of the *Poa* alliance are distributed from the lowlands to the highest mountains as well as from wetlands to semi-deserts. Species of this group are absent from tropical lowlands, but occur in tropical mountains and are especially frequent in temperate to cold zones of both hemispheres. The taxonomy of *Poa* and allied genera is intricate, however, due to molecular studies the character evolution and relationships of this group became clearer during the past years. Many traditionally recognized genera such as *Austrofestuca* and *Dissanthelium* turned out to be nested within a broadly defined and monophyletic genus *Poa* [13], [18], [19]. Genera like *Alopecurus*, *Milium* or *Phleum* with, for example, different inflorescence types or one-flowered spikelets, were previously thought to be distant to *Poa* [20], but are actually close to this genus [13], [16], [18], [19]. The clarification of the evolutionary relationship of *Poa* and neighbouring genera provides first insight into the diversification rates in time and space of this highly diverse plant group with a total of about 775 species. We assembled a data set of 427 internal transcribed spacer (ITS) sequences of nuclear ribosomal DNA representing 217 species of the *Poa* alliance. ITS is by far the most comprehensively studied molecular sequence marker in this plant group. Chloroplast DNA studies using different markers [13], [16], [19] corroborate the relationship of genera and species used in this analysis, still taxon sampling density for chloroplast DNA sequences is too low for diversification rate estimates.

## Materials and Methods

427 nuclear ribosomal ITS1–2 DNA sequences of *Poa* and allied genera, as well as four sequences of outgroup taxa were assembled, representing 217 species. Most sequences were already included in other studies and were collected from GenBank. We added 34 new sequences. These are from insufficiently studied genera or from genera occurring in remote areas (e.g. *Catabrosella*, *Libyella*, *Limnas*, *Coleanthus*, Table S1). Some of these genera were affiliated with the *Poa* alliance but molecular evidence was lacking. Laboratory details are outlined by Schneider et al. [16], [21]. The DNA of the newly included taxa could be sequenced directly, no polymorphisms were detected. *Pholiurus pannonicus* showed in a previous study [21] a few polymorphisms in the ITS region, however, all clones formed a strongly supported clade in the phylogenetic analysis. Two out of the ten sequences were included in our study. GenBank accession numbers and sources of the DNA sequences are available from Table S1. Sequences were aligned using Prank [22] and manually adjusted. Three regions of the ITS sequences (53–55, 194–198, 429–444) that could not be unambiguously aligned were excluded from the phylogenetic analysis. The aligned data set with a length of 623 bp was analyzed by maximum likelihood using RAxML with the GTR substitution model, the CAT approximation of among-site substitution rate variation as well as the rapid bootstrap option and automatic halt as described in the program manual [23], [24] on the CIPRES Science Gateway v.3.1 (www.phylo.org/sub_sections/portal/).

The ITS sequences provide the most comprehensive molecular data set for the *Poa* alliance. Many chloroplast DNA sequences are also available, but they are from different regions of the chloroplast genome and do not enable a dense coverage of taxa. The substitution rates of the ITS vary among groups of the plant kingdom and may be dependent from their life history [25]. Our study group belongs to only one life history type (annuals and perennials), thus ITS sequences may be suitable to study the diversification of the grasses [25]. Molecular dating was performed using the semi-parametric method based on penalized likelihood [26] as implemented in the function ‘chronopl’ of the R package ‘APE v. 2.8’ [27]. The nucleotide diversity [28] and its variance were calculated with the R package ‘PEGAS’ [29]. Unambiguous fossils of the *Poa* alliance are unfortunately missing, thus we had to apply a secondary calibration of our data. Absolute ages of the clades were calculated by fixing the split between the outgroup taxa *Phalaris* and *Avena* and the ingroup of 20 and 25 Myr, respectively. These dates were taken from a dated chloroplast DNA phylogeny of the Poaceae [30], which uses the earliest fossils of the grass family for time estimates. Because not all genera were included in that analysis, an uncertainty remains about the age estimates, but this is alleviated by the application of two ages for the basal node of *Poa* and its allied genera. The node age of 20 Myr is the split between the subtribe Poinae and the other taxa of the tribe Poeae, the conservative age estimate of 25 Myr is from the split between Poeae and Aveneae [30]. The 95% confidence intervals of the lognormal prior for the conservative 25 Myr age estimate of the BEAST analysis (see below) were used to infer confidence limits of the age estimates.

Divergence time estimates were also performed using the Bayesian uncorrelated relaxed clock method as implemented in BEAST 1.6.1 [31] with the conservative 25 Myr calibration point. A lognormal prior using a zero offset and a standard deviation of 0.05 (95% confidence interval 21.1 and 29.3 Myr, respectively) were used. Three independent BEAST runs were performed for 1,000 million generations each, using a birth–death prior for branching rates. The convergence of runs and the appropriate burn-in were assessed using Tracer [32].

**Figure 1:**
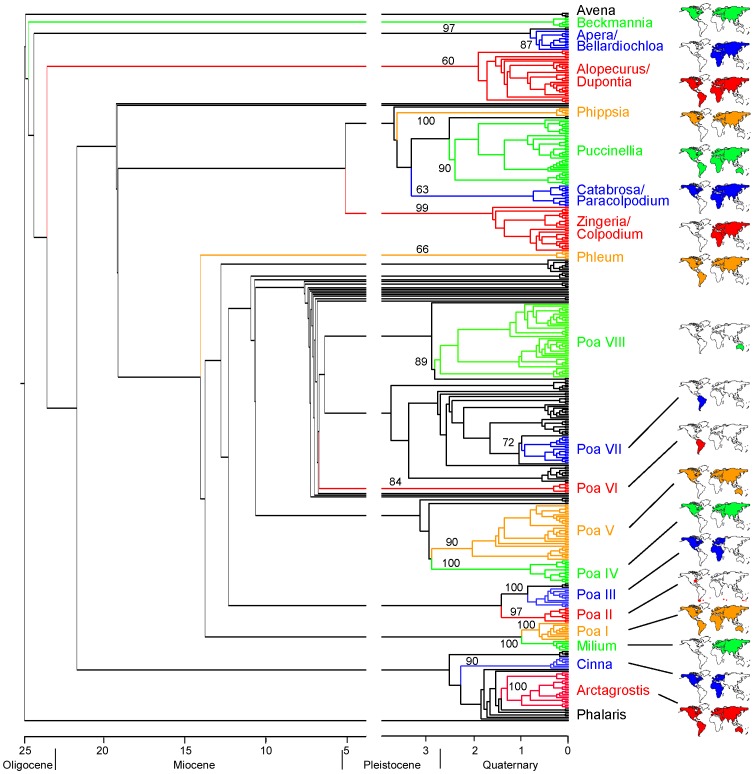
Simplified phylogram from maximum likelihood analysis of ribosomal ITS sequences in *Poa* and related genera. Bootstrap support for the major clades are indicated at the branches. Informal names of the clades are given in conjunction with their distribution. For taxon names see the phylogenetic tree in the figure S1.

Diversification rates were estimated using the method of Magallon & Sanderson [33] as implemented in the R package ‘GEIGER’ [34]. Because the species number of the clades is necessary for calculations of diversification rates, two estimates were calculated. The conservative estimate of diversification rate is based solely on the species number included in the analysis. This assessment is probably overly conservative and underestimates the diversification rates, because particularly in the genus *Poa* taxa are missing from the phylogenetic reconstruction. Nevertheless, the numbers derived from this approach were used in the text. The second approach considers the fact that some genera were completely, but others still incompletely sampled for sequence data. The species numbers of the smaller genera, like *Milium*, *Zingeria* or *Puccinellia* are known and based primarily on Watson & Dallwitz [35], the online database ‘GrassBase’ [36] or the molecular phylogenetic studies, from which the DNA sequences were taken (Table S1). To account for the missing species particularly in *Poa* an estimation of the clade's species numbers are necessary (traditional taxonomy does not help, because sections and other formal groups are frequently para- or polyphyletic). Maximum species numbers of the eight *Poa* clades were estimated under the assumption that the *Poa* taxa were more or less evenly sampled across the clades. The maximum species number of the clades is estimated using the ratio: Number of molecularly studied species of the clade * 500 total species of *Poa*/169 molecularly studied species of *Poa*. Distribution data were obtained from GrassBase [36]. Non-native occurrences were omitted from the maps (figure 1).

## Results

**Figure 2:**
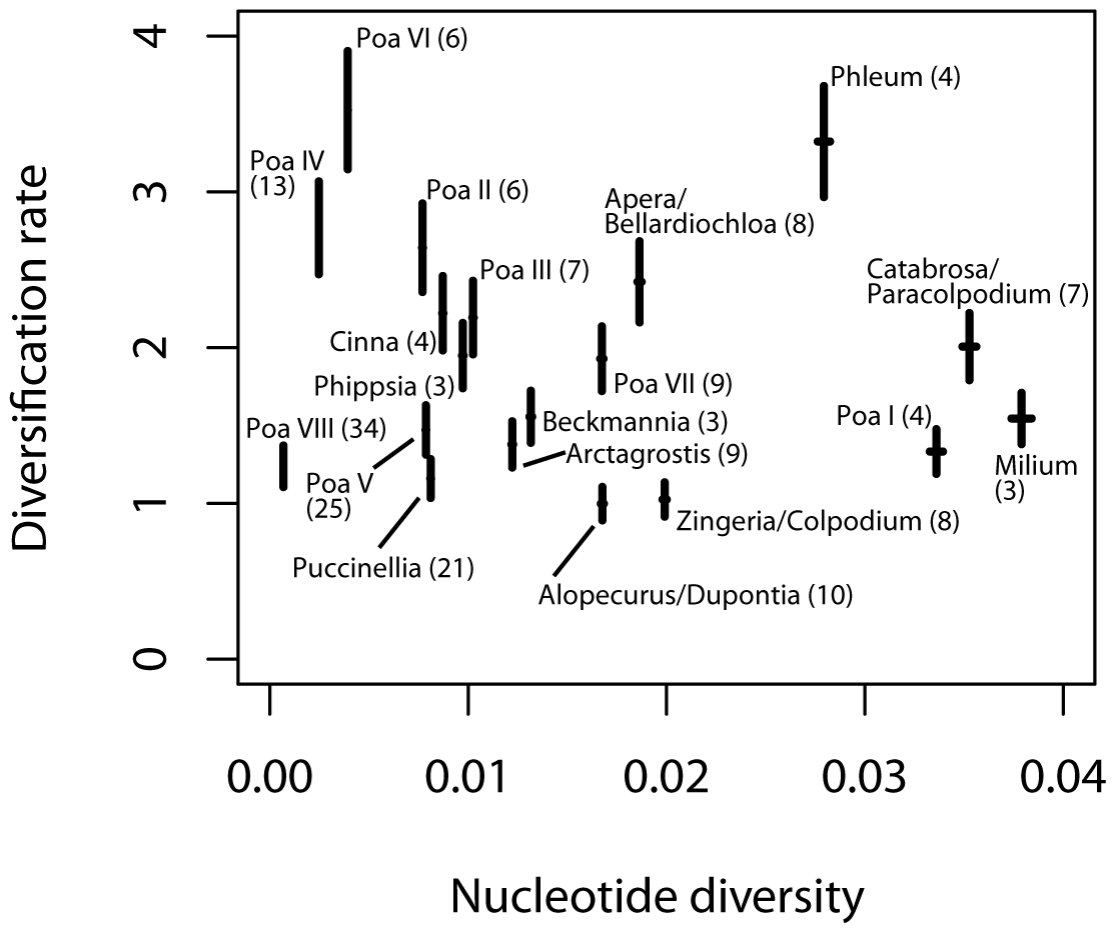
Diversification rates and nucleotide diversity. Estimates of the diversification rates of the clades (figure 1) under the assumption that all taxa of the clades were included in the analysis. The span of the vertical bars refers to the two dates (20 and 25 Myr, respectively) used for calculation of the absolute ages. The lower end represents the diversification rate for the dating with 25 Myr, the upper for 20 Myr. The x-axis indicates Nei's nucleotide diversity of the clades (the numbers in brackets refer to the number of DNA sequences used for its calculation). The variance is indicated as horizontal bars. There is no significant correlation between diversification rate and the nucleotide diversity.

The phylogeny of *Poa* and its relatives shows several well-supported clades that are partly restricted to continents, but sometimes more widespread (figure 1, figure S1, see below). The genus *Poa* in its traditional circumscription is not a monophyletic group. Although it premature to speculate on the future taxonomic treatment of the *Poa* alliance, it seems clear that either some genera may have to be included in *Poa* to extend the genus or *Poa* must be split into a couple of smaller genera. Therefore, we cannot consider the diversification of *Poa* as a whole but have to focus on the rather well-supported clades of the *Poa* alliance. Nucleotide diversity of supported clades is rather low (0.0007 to 0.0379; Table 1, figure 2) and suggests a rapid radiation and recent diversification of the *Poa* alliance.

**Table 1:** Species numbers, nucleotide diversity, clade ages and diversification rates.

						20 Myr	20 Myr	20 Myr	25 Myr	25 Myr	25 Myr	25 Myr	25 Myr	25 Myr
Clade name	Minimal species number *	Maximal number of species	Number of sequences	Nucleotide diversity	Variance of the nucleotide diversity	Age of the clade (Myr)	DR with minimal species number	DR with maximal species number	Age of the clade (Myr) PL	95% CI	Age of the clade (Myr) URC	95% HPD	DR with minimal species number	DR with maximal species number
Alopecurus/Dupontia	10	46	32	0.0168	0.000076	1.45	1.11	2.17	1.81	1.52–2.12	2.85	1.49–5.10	0.89	1.74
Apera/Bellardiochloa	8	18	12	0.0186	0.000105	0.51	2.70	4.28	0.64	0.54–0.75	2.41	0.72–6.98	2.16	3.43
Arctagrostis	9	9	22	0.0122	0.000044	0.98	1.54	1.54	1.22	1.03–1.43	0.83	0.50–1.13	1.23	1.23
Beckmannia	3	3	6	0.0132	0.000068	0.23	1.73	1.73	0.29	0.25–0.34	1.90	0.31–3.83	1.39	1.39
Catabrosa/Paracolpodium	7	14	13	0.0353	0.000350	0.56	2.24	3.47	0.70	0.59–0.82	2.53	1.36–3.86	1.79	2.78
Cinna	4	4	7	0.0087	0.000030	0.28	2.48	2.48	0.35	0.30–0.41	0.48	0.23–0.77	1.98	1.98
Milium	3	5	6	0.0379	0.000507	0.24	1.71	3.87	0.29	0.25–0.34	2.70	1.34–3.25	1.38	3.12
Phippsia	3	4	5	0.0097	0.000043	0.19	2.17	3.72	0.23	0.20–0.28	0.77	0.28–2.10	1.74	2.97
Phleum	4	16	5	0.0279	0.000309	0.19	3.70	11.10	0.23	0.20–0.27	2.46	0.80–5.71	2.97	8.90
Poa I	4	12	11	0.0336	0.000330	0.47	1.49	3.84	0.58	0.49–0.68	1.91	0.92–3.12	1.19	3.07
Poa II	6	18	9	0.0077	0.000022	0.37	2.95	5.89	0.47	0.39–0.55	0.63	0.30–1.15	2.36	4.71
Poa III	7	21	12	0.0102	0.000035	0.51	2.45	4.59	0.64	0.54–0.75	1.12	0.54–1.76	1.96	3.67
Poa IV	13	38	14	0.0025	0.000003	0.61	3.09	4.86	0.76	0.64–0.89	0.44	0.22–0.69	2.47	3.89
Poa V	25	74	34	0.0079	0.000019	1.54	1.64	2.35	1.92	1.62–2.26	0.84	0.53–1.15	1.31	1.88
Poa VI	5	15	6	0.0039	0.000008	0.23	3.93	8.64	0.29	0.25–0.34	0.45	0.33–0.72	3.14	6.92
Poa VII	9	27	16	0.0167	0.000082	0.70	2.15	3.72	0.87	0.74–1.02	1.38	0.38–2.63	1.72	2.98
Poa VIII	34	101	45	0.0007	0.000000	2.05	1.38	1.91	2.56	2.16–3.01	0.79	0.38–1.32	1.11	1.53
Puccinellia	21	107	40	0.0081	0.000020	1.82	1.29	2.19	2.27	1.92–2.67	1.44	0.64–2.29	1.03	1.75
Zingeria/Colpodium	8	12	27	0.0199	0.000107	1.21	1.14	1.48	1.52	1.28–1.78	2.15	0.81–3.37	0.91	1.18
	* This equals the number of species in the analysis													

Columns named with 20 Myr or 25 Myr refer to the analysis using basal node ages of 20 Myr and 25 Myr, respectively. Abbreviations: DR Diversification rate, PL Penalized likelihood, CI Confidence interval, URC Uncorrelated relaxed clock, HPD Highest posterior den.

The molecular dating using penalized likelihood and the uncorrelated relaxed clock method gained comparable age estimates of the clades putting considerable confidence in the age estimates of the *Poa* alliance (Table 1). For 13 out of the 19 assessed clades the 95% confidence intervals overlap. The differences are not restricted to either particularly young or old clades as well as not necessarily to neighbouring clades. In two cases the uncorrelated relaxed clock method provided younger and in four cases older time estimates than penalized likelihood (discussed below). Due to the convergence of age estimates the data from the penalized likelihood analysis is subsequently used in the text if not otherwise stated.

The estimated ages of the crown groups are rather insensitive to the age of the stem nodes. The clade comprising most *Poa* species is estimated to be 10.9 Myr old for a supposed basal node age of 25 Myr and 8.8 Myr for a basal node age of 20 Myr (Table 1, figure 1), which is compatible with other genus and species level studies in related lineages of grasses [30], [37]. Table 1 shows the results from the molecular clock age estimates for the diversifications of the different clades highlighted in figure 1. They split between about 2.1–0.19 (basal node age 20 Myr) and 2.5–0.23 Myr (basal node age 25 Myr), respectively (Table 1), which means a rather recent, Pliocene to Pleistocene diversification. The time frame is in the order of the diversification found for the subtribe Loliinae, a sister clade of the *Poa* alliance [37].

We observed some well supported clades within the *Poa* alliance that are shortly characterized particularly with respect to the ecology and distribution of the taxa. Focus is also set on the life cycle differences of the species, as well as morphological peculiarities of the panicles and spikelets.

### Beckmannia

This basal clade comprises morphologically quite varied taxa. The inflorescence of annual *Pholiurus* is a simple spike comprised of two-flowered spikelets. The inflorescence of the annual or perennial *Beckmannia* is a one-sided panicle whose branches appears to be a one-sided spike, i.e. the one- or two-flowered spikelets possess very short pedicels. All species grow in open conditions. The morphological disparity of this clade is paralleled in rather wide confidence intervals of the age estimates between about 0.3 and 3.8 Myr.

### Apera/Bellardiochloa

Diversification of this species group may have similar as in *Beckmannia*. The species of this clade have open panicles, *Apera* comprises few annual species with one-flowered spikelets. The species are weeds of lowlands and were widely distributed by humans. *Parvotrisetum* comprises annual species with mostly few-flowered spikelets. *Bellardiochloa* consists of two perennial species that are distributed in high mountain areas. The spikelets have also few flowers.

### Alopecurus/Dupontia

According to the two molecular clock estimates this clade is older than the above-mentioned clades (Table 1). The diversification of this clade may have started around the Pliocene/Pleistocene boundary. *Alopecurus* and *Limnas* have condensed panicles and one-flowered spikelets. Perennial *Limnas* comprises only two species that are confined to the boreal parts of Siberia and the Russian Far East. Genus *Alopecurus* is much more diverse with about 50 perennial and annual species distributed in the northern hemisphere, Africa and South America. The species grow apparently always in open conditions. The species seem to have diversified particularly along a soil moisture gradient from almost boggy places (*A. geniculatus*) to mesic habitats (*A. pratensis*). Temperature is another gradient along which the species are differentiated. There are species confined to lowlands (*A. pratensis*) or high mountains (*A. glacialis*, *A. stejnegeri*), of southern areas or arctic environments (*A. alpinus*). In comparison to *Phleum*, which has similarly condensed panicles, *Alopecurus* appears not to grow in too dry soils. *Arctophila* and *Dupontia* are almost confined to the Arctic. *Dupontia* is probably of hybrid origin and *Arctophila* is involved in this origin [38]. Both genera comprise perennial species that have open to condensed panicles and few-flowered spikelets. According to the ITS sequences, *Limnas* may also be involved in the origin of *Dupontia* as the second parent. The sometimes strongly condensed, spike-like panicles of *Dupontia* support this suggestion [20].

### Arctagrostis and Cinna

The widespread clade corresponds to the ‘HSAQN clade’ [39]. It comprises perennial species with open panicles. The spikelets of the plants belonging to this clade are sometimes one-flowered (*Cinna*, *Arctagrostis*) but usually many-flowered. In terms of the ecological differentiation the whole clade shows an astonishing diversity, because species occur in temperate forests of Australia and the northern hemisphere (*Sylvipoa*, *Cinna*), temperate high mountains habitats (*Saxipoa*, *Poa* sect. *Arctopoa*), or even in arctic environments (*Arctagrostis*). The diversification of these plant groups may have started less than 1 My ago.

### Zingeria/Colpodium

The basal species of this clade (*Catabrosella araratica*, sometimes considered to be a species of *Colpodium*) has few-flowered spikelets, whereas the other species of this clade have one-flowered spikelets that are arranged also in an open panicle. All species are perennials. The caryology of *Catabrosella araratica* is still unknown, the other species are characterized by rather few but large chromosomes. The reduction of chromosome numbers and a subsequent polyploidization was perhaps the key trait for the diversification of this clade [40]. All species occupy open and mesic to moist habitats from the lowlands to higher mountain areas. The species of *Zingeria* seem to grow frequently also in disturbed places.

### Phippsia

This clade comprises the two perennial species of *Phippsia* and the annual *Coleanthus subtilis*. Common features are one-flowered spikelets and an open panicle. The species of *Phippsia* are almost confined to the Arctic, whereas *Coleanthus* occupies a scattered range across the northern hemisphere. The diversification of this clade may have started less than 1 Myr ago and thus well beyond the formation of the circumpolar arctic tundra ecosystem.

### Puccinellia

*Puccinellia* is an apparently monophyletic and almost world-wide distributed genus comprising about 80–120 species. The genus may have started its diversification about 2 Myr ago and gained in this rather short time an astonishingly high diversity in terms of life form and ecology. Most species are perennials, a few are biennials or short-lived. The spikelets are more than one-flowered. Many polyploids are known in this genus [41]. Most if not all species grow in open conditions from dry to wet soils. A prominent ecological trait of *Puccinellia* is the comparatively high salt tolerance of the species that may have enabled the genus to diversify rapidly. The species are strongly diversified along an altitudinal, latitudinal and longitudinal gradient predominantly in salty soils.

### Catabrosa/Paracolpodium

The species of this clade are perennials and comprise open panicles. *Hyalopoa* and *Catabrosa* have few-flowered spikelets, whereas *Paracolpodium* has only one-flowered spiklets. The species of *Hyalopoa* and *Paracolpodium* occupy mostly mesic high mountain habitats, whereas *Catabrosa* prefers bogs or other wet places. In this clade the methods of age estimation differ slightly. Penalized likelihood revealed a younger age, whereas the uncorrelated relaxed clock method date the diversification earlier. The borders of the confidence limits are about 0.5 Myr apart.

### Phleum

The very close relationship of *Poa*, *Milium* and *Phleum* was hardly supposed on morphological reasons. *Milium* and *Phleum* possess one-flowered spikelets with a short terminal appendage that are arranged in *Phleum* in a condensed panicle. Based on the varying degree of incorporation of inflorescence branches into the inflorescence axis some sections were distinguished. Most of them comprise annual as well as perennial species. The annual species are mostly confined to the Mediterranean region and seem to prefer dry places. Perennial *Phleum* species show an ecological differentiation. *Phleum phleoides* is widespread on dry soils in the lowlands, whereas *P. pratense* occupies moister soils in the lowlands. *Phleum alpinum* and *P. hirsutum* occur mostly in mountains, the former species prefers generally acidic or neutral soils, whereas the latter grows mainly on calcareous soils. This rather strong ecological differentiation that perhaps drove the diversification of *Phleum* is paralleled in the Poa IV clade and possibly in the Poa V clade.

The penalized likelihood method estimated *Phleum* and the below-mentioned *Milium* and *Poa* I clades about 2 Myr younger than the method implemented in the BEAST software. These clades are seemingly related, however, the bootstrap supports of the clades are low. The nucleotide diversity of these three groups as well as that of the *Catabrosa*/*Paracolpodium* clade are highest in our sample (Table 1, Fig. 2). At least two explanations may be possible for this phenomenon and disparity between the methods. First, in accordance with the higher genetic differentiation among the species the uncorrelated relaxed clock method places the time of divergence appropriately more back in time. Secondly, the molecular evolution proceeded with higher rates in the 4 clades and the results of the penalized likelihood method reflect this accordingly. More molecular analysis that strengthen the backbone of the tree are necessary to decide between these possibilities.

### Milium

The small genus *Milium* comprises annual as well as perennial species of Eurasia that have only one fertile floret in the spikelet and sometimes a short terminal appendix. *Milium* has been placed in several groups of the grasses. An affiliation with the *Poa* alliance has been suggested sometimes already on morphological reasons (e.g. [20] and was subsequently corroborated by several molecular studies [13], [16], [19], but see [14] for a different position). One-flowered spikelets are occasionally found also in Malesian and South American *Poa* species and may be the result of a parallel reduction of flowers.

### Poa I

This group of annual and short-lived perennial species has been collated together with some species of the Poa II and III groups into section or subgenus *Ochlopoa* that do not appear to be monophyletic. The species were mainly found growing in open conditions, *Poa annua* is a world-wide distributed weed.

### *Poa* II

This clade comprises a few perennial species, which are distributed mainly on the subantarctic islands, southern South America and New Zealand. A prominent characteristic of these species is their rather condensed panicles with only short branches. North American *P. chapmaniana* is deviating from the other species of this group, because it is annual and does not have a condensed panicle. The diversification of this group may have started in the Holocene.

### *Poa* III

Most species of this somewhat older clade occur in Europe and are characterized by their densely cespitose growth form and the frequent formation of a bulbous stem base. Some species, for example the widespread *P. alpina*, have proliferating spikelets. The species are generally found in open and usually somewhat dry conditions.

### *Poa* IV

Probably also of Late Pleistocene/Early Holocene origin are the species of the *Poa* IV clade that were affiliated with different sections (e.g. *Stenopoa*, *Abbreviatae* [20]) and are widespread in the northern hemisphere. All species are perennials with a tufted growth or are creeping with stolons. Usually the species of this clade are widespread and highly diversified in terms of their ecological preferences. They occur in open (*P. compressa*) or forested areas (*P. nemoralis*), some species prefer moist to swampy soils (*P. palustris*) or dry habitats (*P. botryoides*). The species are found from the lowlands (*P. palustris*) to the alpine belts (*P. glauca*) and from the temperate (*P. nemoralis*) to the arctic zones (*P. hartzii*).

### Poa V

This group comprise two weakly supported sub-clades. One sub-clade consists of widely distributed perennial species of the northern hemisphere and comprises additional species, which reach the Arctic (e.g. *P. arctica*). According to Tzvelev [20] these species belong to subsect. *Poa*. This affiliation is not supported in our phylogenetic tree. The other sub-clade, named ‘X-clade’ [39] comprise endemic and perennial species of New Zealand. These New Zealand species are enigmatic in terms of their incongruence between nuclear and chloroplast markers [39]. Most species have bisexual florets, *P. novae-zelandiae* is gynomonoecious and a couple of species are dioecious (*P. foliosa*, *P. subvestita*, *P. sudicola*, *P. xenica*). The species of this group appear to have diversified into a variety of habitats to which they are confined [42]. Most species grow in open conditions predominantly in the subalpine to the alpine zone, but also on exposed cliffs and rocks. Some species grow in open forests, e.g. *P. matthewsii*. Many growth forms are represented in this New Zealand group: almost tussock forming species to slender rhizomatous species. Considering the rather frequent bipolar disjunctive distributions between northern Asia and Australia/New Zealand (e.g. *Carex pumila*) it seems likely that this group diversified ecologically and morphologically rapidly in New Zealand after its arrival from the North. This ecological diversification seems to be paralleled in the Poa IV clade on the northern hemisphere.

Not resolved among the two subclades is *P. leptocoma* from the amphi-Beringian region, which was placed in sect. *Oreinos* [20]. Species of this section occur in several places of the phylogenetic tree. This clade is estimated to be between 1 and 2 Myr old, the two dating methods differ. Penalized likelihood gained slightly (0.8 to 1 myr) older age estimates.

### *Poa* VI

This is a small group of perennial high Andean species that were affiliated with different taxonomic groups (*Homalopoa* and ‘*Punapoa*’, [19]). It diversified probably less than 500,000 years ago. *Poa lepidula* was formerly placed in the monotypic genus *Anthochloa* due to its flabellate lemmas [18].

### *Poa* VII

The species of this small clade were unified under the separate genus *Dissanthelium*. The dwarf plants are confined to the Andes of South America, only one species extends northwards to Mexico [19]. The diversification of this group started about 1 Myr ago. A quite uncommon feature of these annual and perennial plants are the gynomonoecious spikelets with usually two florets, i.e. the lower floret is bisexual and the upper florets is pistillate.

### *Poa* VIII

This is a species-rich clade of Australia and New Zealand, which was recognized as a recently diverged group (as sect. *Brizoides*, [39]). The clade consists of mainly perennial species (only *P. fax* is an annual). As in other clades, the species are highly differentiated in terms of their ecological and geographical distribution [43]. Centre of diversity of this clade are the coastal and mountain areas of south-eastern Australia and Tasmania. Besides the species occurring in New Guinea (*P. keysseri*), New Zealand (*P. anceps*, *P. cita*, *P. cockayniana*, *P. intrusa*) or the southern Pacific Islands (*P. chathamica*, *P. litorosa*) only a few species occur in south-western Australia (e.g. *P. homomalla*, *P. porphyroclados*). Only *P. poiformis* and the annual *P. fax* are widespread and occur more or less throughout the southern margin of Australia. As in other clades of *Poa* the species are ecologically highly differentiated. Most species grow in rather dry soils, however, *P. clelandii* grows in moist to wet soils predominantly in lowlands, *P. costiana* in high mountain moist places. Species of woodlands are rare (e.g. *P. sieberiana*, *P. ensiformis*), most species grow in open conditions. The only species occurring in the drier inland of south-western Australia is *P. fordeana*, all other Australian species of this group occupy more or less areas close to the coast. Like in the *Poa* V clade the penalized likelihood estimate of divergence time is about 0.8 Myr older than that of the uncorrelated relaxed clock method. Considering the lowest molecular divergence between the species observed in this study may favour the younger age estimates.

### General patterns of diversification

A frequent pattern in the rapid diversification of the *Poa* alliance is the speciation along ecological gradients. This becomes particularly evident in the following clades: Poa IV, Poa V, Poa VIII, *Phleum*, *Puccinellia*, *Alopecurus/Dupontia*, *Arctagrostis* and *Cinna*. The diversification of these groups has led to species that are ecologically specialized und thus spatially more or less clearly separated from each other. Morphological characters, which may have enabled this rapid diversification, are hardly discernable. Changes in the life span, i.e. annual or perennial growth were probably not the key innovation for radiations, because some perennial groups diversified very rapidly, whereas some clades comprising annuals do not show an elevated differentiation. Changes in the number of flowers per spikelet also does not seem responsible for the diversification. Condensed panicles are a prominent trait of *Phleum* and *Alopecurus*, but groups with open panicles diversify similarly rapidly.

## Discussion

Earliest unambiguous grass macrofossils date back to the Palaeocene/Eocene boundary (55 Myr [44]). Otherwise, the Tertiary sediments are very poor in grass macrofossils [44]–[46]. The discrepancy between the early fossil record and a subsequent paucity in fossils may be due to a long lag-phase in the family's diversification. In North America open vegetation with grasses appeared probably during the Early to Middle Miocene, in Eurasia even later [47]. Phytolith assemblages suggested that grasses were present during the interim time in woodlands as well as open landscapes [48]. The earliest C_3_ grasses reported from the Miocene of North America were, besides the bambusoid grasses that grow predominantly in the understory of forests, related with *Stipa* [48]. These two lineages, i.e. bambusoid grasses as well as *Stipa* and relatives, belong to be the oldest lineages of the exclusively C_3_-species comprising BEP clade of grasses (BEP is an acronym for the subfamilies Bambusoideae, Ehrhartoideae and Pooideae). *Stipa* supposedly split from the remainder of the Pooideae subfamily around the Eocene/Oligocene boundary [30]. This indicates that some diversification of these C_3_ grass lineages proceeded early and before the onset of the C_4_ grass evolution, which led to extensive formation of open grasslands. According to the molecular clock results, the *Poa* alliance was not yet present during this early formation of grasslands (this study and [30]). Paleontological evidence further suggested, however, that the grass-dominated vegetation became abundant in North America not before the Middle to Late Pliocene [46], when large areas were covered by C_4_ grasses [47]. Our molecular dating suggests that many lineages of the *Poa* alliance evolved not much earlier than c. 7 Myr and the radiations of these lineages started less than 4 Myr ago (figure 1). This time frame coincides with the formation of initially temporal and later permanent ice sheets on Greenland as well as in the Antarctic [49], which marks a significant cooling on Earth. The uplift of many mountains during the Neogene, for example the Cascade Range, the Himalayas and Andes has led to drier conditions in the continents interior or at higher elevations in mountains that became occupied preferentially by C_3_ grasses [50]. It was hypothesized that adaptation to cold and dry areas has been the key for the spread of C_3_ grasses and influenced the balance between C_3_–C_4_ grasses in favour of the C_3_ grasses [50] in the more cold and drier areas of the world. These environmental changes of cooling and aridification as well as geological and paleontological evidence fit in space and time the diversification of the *Poa* alliance.

The average species diversification rates of the clades range from 1.11–3.93 (basal node age 20 Myr) to 0.89–3.14 species per Myr (basal node age 25 Myr), respectively (Table 1, figure 2). Considering that not all species were molecularly studied, the real diversification rates may be considerably higher (Table 1). The diversification rates of the *Poa* alliance are in the same range as that of other radiations [2], [5]–[7]. Multiple lineages in the *Poa* alliance show this parallel and rapid origin of morphologically separated new species, as similarly seen in *Lupinus* [6]. The striking morphological diversification of these grasses is not coupled with a comparable molecular diversification of the neutrally evolving DNA sequences (ITS) used in this study. This means that sequence divergence is not necessarily corresponding with, or a predictor of, speciation rates. A similar pattern of disparity between morphological and molecular diversification has been observed in temperate bamboos [51].

Despite the characters that have led to the description of segregate genera, it is notable that the multiple diversifications in *Poa* and the allied genera are not evidently associated with the acquisition of morphological key characters. For the environmental “island situation” of South Africa ecophysiological key innovations are considered as important prerequisite of rapid plant speciation [3], [11]. Cold and drought adaptation, albeit with many exception, was probably an overall key innovation that has evolved during the diversification of the subfamily Pooideae as a whole and thus prior to the diversification of the *Poa* alliance [50]. Particular ecophysiological key innovations of the latter are not known at present.

The diversification of *Poa* and allied genera proceeded only partly in island-like areas. Two clades with different diversification rates are actually confined to the South American Andes, i.e., the rather species-poor *Poa* VI clade with the highest diversification rate observed in this study and the species-rich *Poa* VII clade, which represents the former genus *Dissanthelium* (figure 1, see also figure S1). According to the molecular clock, the *Poa* VI clade started to diversify 0.05, the *Poa* VII clade about 1 Myr ago (calculated with stem age of 25 Myr). These time estimates are in the order of Andean *Lupinus* or Eurasian *Dianthus* diversifications, which started less than c. 2 Myr ago [2], [5], [6]. The speciation of the *Poa* VII clade proceeded at a slower pace, but had more time available and thus became more species-rich than clade VI (figure 1, 2).

In contrast to these diversifications in the Andes or the Mediterranean/Irano-Turanian regions, the *Poa* II and IV clades with diversification rates of about 2.5–3 species per Myr are not restricted to island-like areas. The *Poa* II clade occurs in North and South America, Australia and New Zealand, whereas the *Poa* IV clade is widespread in the Northern Hemisphere. The latter clade comprises widespread taxa that are highly diversified in terms of their ecological preferences. Two clades of *Poa* (clade VIII, and a less well supported part of clade V), which are restricted to Australia and New Zealand, underwent a slower diversification than the Andean and Northern hemispheric clades. The independent evolution of two lineages in Australia and New Zealand, which arrived probably by long distance dispersal, is paralleled, for example, in the genus *Ranunculus* [52], [53].

The genera related with *Poa* show overall similar diversification rates, but have higher nucleotide diversity (e.g., *Catabrosa*, *Milium*) and appear to be molecularly faster evolving than *Poa* (figure 2). *Phleum*, a genus with peculiar condensed inflorescences, diversifies probably most rapidly among the grass genera studied, whereas *Alopecurus* with similar inflorescences diversifies slowly. The genera with only few and large chromosomes (*Colpodium*, *Zingeria*) have diversified with low rates. None of these genera is restricted to island-like areas.

The global diversification of the bluegrasses and related genera provides an example of rapid and parallel speciation in a wide variety of habitats, continents and areas. Some of these features were singly reported also for other plant genera and families, but here we observe them together in a species-rich group of grasses. The members of the *Poa* alliance are important components of grasslands in either the temperate to arctic zones or the high mountain belts of tropical to subtropical zones. Although some lineages date back to the Middle Tertiary, they started to diversify strongly not before the global cooling changed the environmental conditions fundamentally. Cold tolerance, suggested to be one of the ecological key innovations of the subfamily Pooideae [50], may have been acquired during the early diversification of the subfamily, but became significant millions of years later during the radiation of the *Poa* alliance.

## Supporting Information

Figure S1 **Complete phylogenetic tree of all ITS sequences used in this study.** The colors are as in figure 1, the numbers above the branches are bootstrap support values >50%. The original taxon names as recorded in GenBank were used.
(PDF)

Table S1 Taxon names, GenBank accession numbers and sources of the ITS sequences used in this study.
(XLS)
