# Supplementary figures and images for: Rapid and Recent World-Wide Diversification of Bluegrasses (Poa, Poaceae) and Related Genera
Source: PLoS One. 2013 Mar 27;8(3):e60061. doi: 10.1371/journal.pone.0060061 (PMC3609727; doi:10.1371/journal.pone.0060061)

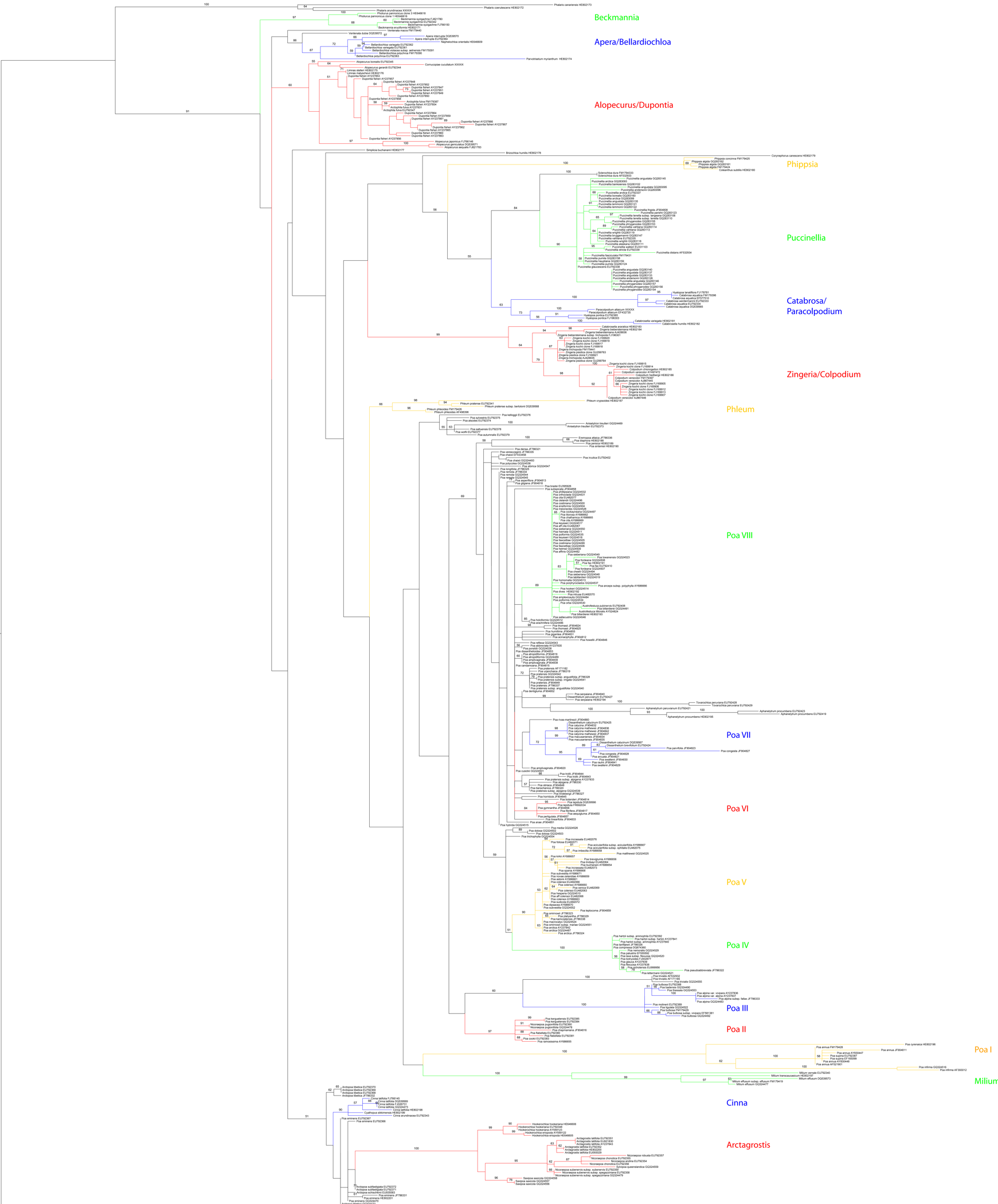

Supplement: Figure S1 — Complete phylogenetic tree of all ITS sequences used in this study. The colors are as in figure 1, the numbers above the branches are bootstrap support values >50%. The original taxon names as recorded in GenBank were used. (PDF) [file pone.0060061.s001.pdf]
